# P90 - Comparing the accuracy of skin prick tests to fresh milk or cow’s milk solution in predicting challenge outcomes

**DOI:** 10.1186/2045-7022-4-S1-P145

**Published:** 2014-02-28

**Authors:** Carol Dungu, James Gardner, Giuseppina Rotiroti, Katrien Coppens

**Affiliations:** 1 Department of Child Health, Royal Free Hospital, London, United Kingdom

## Introduction

Allergy to cow’s milk is the most common allergy found in infants and young children. The estimated prevalence of milk allergy is 2-7.5% (Vandenplas) and the variability relates to diagnosis methods used and services available in any given demographic area.

Skin prick tests (SPTs) are a well established and widely used method of allergy testing for cow’s milk. Many clinics use commercially available solutions in their clinics with the use of some fresh foods but there has been limited research available comparing the accuracy of these solutions compared to fresh foods and a literature search carried out in August 2012 only found 1 paper by Mauro et al. who looked into this.

At the Royal Free Hospital, London UK, we introduced fresh milk SPTs alongside the use of solution in August 2011 to aid our decision making in these children.

## Aim

To compare the accuracy of skin prick testing to fresh cow’s milk and cow’s milk solution in predicting challenge outcomes.

## Method

A retrospective audit of hospital based cow’s milk challenge outcomes was correlated with their skin prick tests results, as documented in clinic letters, during an 18 month period (August 2011 to January 2013). Information was also collated on whether they had other associated food allergies or allergic diseases.

## Results

In total there were 27 patients who had cow’s milk challenges during the 18 month period.

70% (n=19) of this group had a negative solution test, 37% (n=10) a negative fresh milk test and 37% (n= 10) of them had negative tests to both the solution and the fresh milk.

If the patient’s had positives to both solution and fresh milk at least one value was less than 5mm.

### Failed challenges

15% (n=4) of the cohort failed the challenge (75% female) with ages ranging from 4 months to 5 years

Types of reactions included vomiting as an immediate or delayed reaction, urticaria and angioedema.

The skin prick test values to solution ranged from negative to 4mm and for the fresh milk negative to 9mm. The biggest difference between solution and fresh values was 5mm.

75% of the patients who failed the milk challenge had at least one other allergic disease (Asthma, eczema and Rhinitis) or another food allergy but there was 1 patient (25%) who did not have any other allergic disease.

Half of the children who failed had other food allergies, none had Asthma, 25% Rhinitis and 75% had Eczema.

1 patient had 1 other allergic disease, 1 had 2 other allergic diseases, and the eldest child who failed had rhinitis, eczema and other food allergies.

### Passed challenges

85% (n= 23) of our cohort passed the challenge (48% female) ranging from 5 months old to 14 years 9 months old (mean age. 3Y8M)

Solution SPTs ranged from negative to 4mm and fresh values ranged from negative to 10mm.

74% of those who passed the challenge had negative solution SPT versus 39% with negative fresh SPT. In all cases of those who tested positive to both the solution and fresh milk, the lower value was less than or equal to 4mm.

39% of those who passed had both solution and fresh negative SPT. The biggest difference between solution and fresh values was 10mm.

30% of these children had no other allergic diseases or food allergies. 35% had one other allergic disease and 17% had two other allergic diseases.

There was one girl (aged 14Y1M) who had all three allergic diseases (asthma, rhinitis and eczema) and also had other food allergies.

## Conclusion

There was no correlation with the values of the skin prick tests values, solution or fresh, and the outcome of the challenge in our group of patients. As with most aspects of treating allergic patients the history plays the most significant role when deciding on treatment paths and challenges remain the gold standard.

## Future

Repeating the audit with larger numbers of patients could yield a more consistent pattern and guidance for cut off levels.

As allergy testing becomes more sophisticated the use of recombinant provides another method of predicting allergic reactions and it is now a regular part of our clinical practice in addition to skin prick tests.

